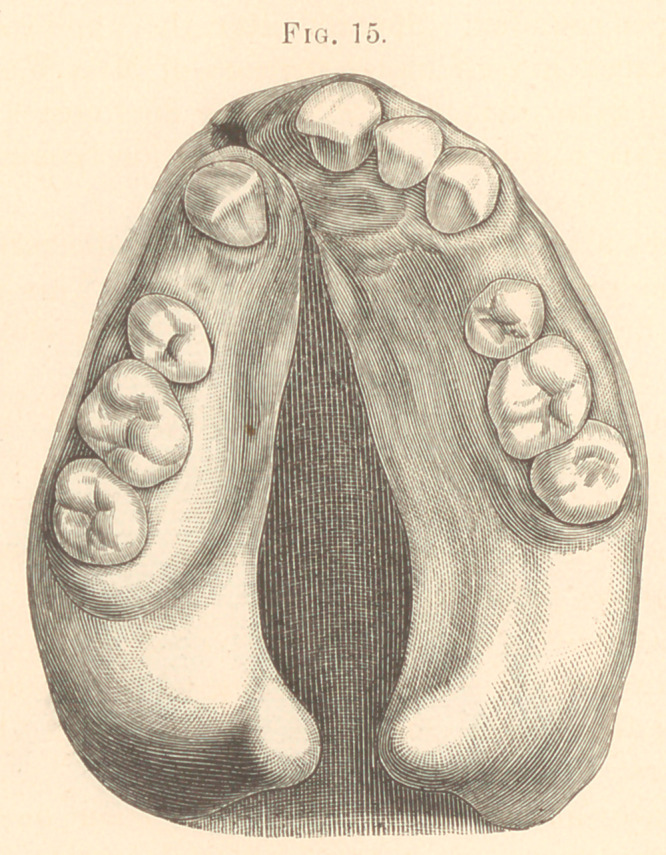# Oral Deformities and Their Correction

**Published:** 1890-07

**Authors:** Kasson C. Gibson

**Affiliations:** New York, N. Y.


### International Dental Journal.

VOL. XI. JULY, 1890. NO. 7.

### Original Communications.[1]

### ORAL DEFORMITIES AND THEIR CORRECTION.[2]

BY KASSON C. GIBSON, NEW YORK, N.Y.

IN the preparation of this paper no attempt has been made to give a history of what has been written on this subject, or a description of appliances used by others, either for the restoration of speech in acquired lesions or for the improvement of articulation in congenital defects; but briefly to describe the methods employed in making and adapting appliances for a few special cases, which have come under my care.

Defects of the palate, hard and soft, are acquired or congenital.

The former are caused by a pathological change of structure (gunshot or missile), and may be divided into three classes:

*First*, Consist of perforation of the vault of the palate.

*Second*, Perforation of the velum.

*Third*, In the entire destruction of the vault of the palate, or a great portion of it. To this last might also be added the destruc-

[1] The editor and publishers are not responsible for the views of authors of papers published in this department, nor for any claim to novelty, or otherwise, that may be made by them. No papers will be received for this department that have appeared in any other journal published in the country.

[2] Specially reported for the INTERNATIONAL DENTAL JOURNAL from a paper read by Kasson C. Gibson, of New York City, before the Maryland State Dental Society, December 5, 1889, on "Obturators for Acquired and Congenital Defects of the Hard and Soft Palate."

tion of the whole or larger part of the velum, as well as the vomer, part of the alveolar border, and turbinated bones.

Congenital defects are the result of malformation or imperfect development of the parts. These occupy the median line, and consist in a division of the osseous and soft textures of greater or less extent.

This division is sometimes confined to the vault of the palate; at other times the velum, the anterior part of the alveolar arch, and the upper lip may be imperfectly developed. It may form a communication with both nostrils, and, when the malformation extends to the alveolar border and upper lip, the latter, divided vertically in one and sometimes two places, gives to the mouth a most disagreeable aspect.

The hare-lip is sometimes met with when there is no imperfection of the osseous structure, and imperfections are often met with here when the lip is perfect.

In some cases the cleft or fissure is more than three-fourths of an inch in width throughout the whole extent of the palate and velum, extending through the whole of that portion of the alveolar border which should be occupied by four incisors; at other times the alveolar arch is divided in two places, leaving a portion between the lateral and central incisors, or one lateral and one central incisor, which, projecting more or less, greatly increases the deformity.[1]

Although a double hare-lip with two divisions of the alveolar border is seldom met without some defect of the palatine organs, cases do occasionally occur.

Congenital defects of the palate are sometimes accompanied by more or less deformity of the sides of the alveolar arch and of the teeth.[2]

---

[1] This classification of acquired and congenital defects of the palatine organs is after Delabarre.

[2] In this connection, it is deemed appropriate to present the following cases of compound complicated hare-lip with cleft palate, which were operated on by Professor James L. Little, of New York City. These cases are of peculiar interest, due to the fact that all were complicated, occurring in the same family, and were not operated on until early manhood.

I quote the following from Dr. Little's pamphlet:

"These cases will be described in the order in which they came under my observation.

"William Bocock, aged twenty-one.

"John Bocock, aged nine.

"Charles Bocock, aged eighteen.

When the fissure extends through both the hard and soft palate no benefit is derived in articulation from staphyloraphy; where the soft palate only is involved this operation is rarely successful so far as articulation is concerned, as the palate is contracted, bringing it forward and too greatly increasing the space between it and the pharynx.

In both congenital and acquired cases the principal effects resulting from the absence of a portion of the palatine arch are impairment of the functions of mastication, deglutition, and speech. In the former the habit of breathing through the mouth is often added, and consequent otalgia.

The last fact was brought to my notice about ten years ago by Dr. D. B. St. John Roosa, aurist, of New York, who advised the wearing of an obturator, even if normal breathing were the only benefit derived. The following case is illustrative:

In March, 1882, Miss A., aged about thirty, having congenital fissure of the soft palate, and a mouth-breather, had suffered from childhood during the winter months with earache, and in consequence had almost totally lost her hearing. She was advised to have an obturator; this was constructed of hard rubber. The

---

"No hereditary tendency can be traced in father's or mother's family. There were four boys and five girls. All the boys were born with hare-lip, while no deformity existed in any of the girls.

"The order in which the children were born is as follows:

"1. William: Compound complicated hare-lip.

"2. Girl with no deformity.

"3. Charles: Compound complicated hare-lip. A spindle-shaped sarcoma made its appearance on the left side of the perineum in 1878, which I removed. It recurred, and I again removed it in 1882.

"4. Girl with no deformity.

"5. Girl with no deformity.

"6. John: Compound complicated hare-lip. Absence of ring finger on right hand.

"7. Girl with no deformity.

"8. Girl with no deformity.

"9. Boy with single hare-lip, who died in infancy.

"These patients presented this deformity in almost the worst form possible, the arrest of development occurring at a very early period of fœtal life.

"The inter-maxillary bone in each case was distinct, being ununited to the superior maxillaries, and was continuous with the nasal septum and vomer. The projecting bone was partially covered by a tag of integument which was continuous with that of the tip of the nose.

"In John (Case 6) the bone contained two well-developed incisor teeth, while in William and Charles (Cases 1 and 3) there was but one.

patient, after wearing the appliance seven years, stated she had been relieved of earache, but her hearing had not improved. It was a surprising fact that her articulation had become nearly perfect notwithstanding her inability to hear her own voice.

A young man with a congenital fissure of the soft palate, who had been wearing a hard rubber obturator for four or five years, met with an accident which necessitated the repairing of a clasp. Failing to call at the appointed time, he gave as his excuse, on calling several days after, that he had caught cold and had been suffering from earache, the first time since wearing the appliance. Unknown to me he had been afflicted with earache from childhood. At his suggestion a duplicate of the obturator was made for use in an emergency.

In these cases hard rubber, being a non-conductor, should always be used instead of metal.

In acquired lesions, where the uvula and velum have been destroyed and a properly-fitted obturator substituted, the readiness with which articulation is restored is due largely to the more developed power of the tongue, also of the constrictor muscles of the pharynx.

On the other hand, congenital fissures of the palate present far greater obstacles, it being very difficult to adapt an artificial organ so that power is given to acquire perfect speech, when from defect of the natural organ the patient from birth has been incapable of distinct utterance.

---

"There was a complete absence of both the hard and soft palate in all three cases, and in Case 6 the fissure was unusually wide (4 centimetres).

"Articulation was so imperfect that they could be understood with the greatest difficulty. . . .

"In concluding this paper I desire to say a few words regarding uranoplasty and staphylorrhaphy. I had performed these operations successfully before operating upon the case described in the first part of this paper. Since that time I have carefully looked into the results and find that although in a large proportion of the cases the operations are successful so far as the closure of the fissure in the hard and soft palate is concerned, yet so little, if any, benefit is obtained in the improvement of the articulation that I have been forced to the conclusion that they should be discarded as surgical procedures in adults. I refer, of course, to cases in which the cleft is congenital. Mr. George Pollock says, 'The real object of the operation of closing the cleft in the palate is to enable the patient to articulate hereafter plainly and intelligibly,—*not* to enable the child to take food.'"

It has recently been brought to my notice that a boy born to the eldest girl in the family has a compound complicated hare-lip, the exact counterpart of Case 3.

It is possible, even in these cases, after an obturator has been properly adjusted, to so educate the tongue and constrictor muscles of the pharynx that they will be able to perform functions, they never would have been required to exercise in conjunction with perfectly-developed organs.

The successful results, however, depend largely on the intelligence and efforts of the patient to learn to articulate properly.

Obturators have been made for congenital fissures, enabling the wearer to articulate with distinctness.

Lessons in elocution will prove of great assistance to the patient. The method employed in teaching deaf mutes to articulate has been applied to these cases with satisfactory and more permanent results.

In simple cases of congenital fissures, correct articulation is acquired more readily than in complicated ones. In the latter, when a defective lip, enlarged nostril, or both, exist, combined with a deformity of the hard and soft palate and very often with imperfect occlusion of the teeth, the difficulties are greatly augmented.

With these abnormal conditions the construction and adaptation of an appliance becomes proportionately difficult.

In view of these facts it is not advisable to always hold out positive assurance of success.

Obturators prove of great benefit in other ways even if improvement in articulation is not marked.

They should, if possible, be secured by clasping, thus preventing any possibility of the appliance becoming loose and dropping into the œsophagus. For the natural relationship of the parts, see frontispiece.

Figs. 1 and 2 of plate are from plates taken from the "Atlas of Topographical Anatomy," by Wilhelm Braune, Professor of Anatomy, in the University of Leipsic.

These figures show the organs of speech in their true normal position, and a careful study of them and their relative positions to one another will greatly facilitate the proper adapting of an appliance.

Fig. 1 was taken from the body of a powerful, well-built, perfectly-normal man. The organs exhibited no pathological irregularities. The body, which was brought in unfrozen, was placed on a horizontal board without any special support for the head. In this position the subject lay in the open air at a temperature of about 50° F. for fourteen days. At the end of this time the process of freezing was completed.

The mesial line of the body was next accurately marked out anteriorly and posteriorly with a black line, and the section carefully performed by means of a fine-edged saw, much in the same way as two workmen would saw the trunk of a tree.

Fig. 2 was a section made on the body of a finely-formed woman, which was brought into the dissecting-room immediately after death. The arteries were injected with paint and the body laid on the back and frozen and the details of the section carried out as in the first case.

The following cases are instructive as to the treatment adopted in their correction.

Female, aged thirty-five. Syphilis acquired. Perforation about the size of a pea, near the centre of the upper jaw left of the median line. All the teeth had been extracted except the central incisors, second bicuspid, second molar on left side.

Previous to taking impression in plaster of Paris, the tissue about the perforation was wiped dry, a piece of adhesive plaster about the size of a five-cent piece was accurately fitted to cover the perforation, thus preventing plaster from passing through.

After adjusting, this was oiled to prevent the plaster of Paris from adhering.

A plate was made of rubber covering the perforation, but not extending into it with teeth to supply the ones which had been extracted. If the lesion due to disease is simple, the plate or obturator should bridge across, but not extend into perforation. If the uvula and velum are destroyed, the obturator should be fitted anterior or posterior to the opening.

In all cases due to disease the appliance should be so constructed and adjusted as to avoid irritation.

1872. Boy, aged eleven. Hereditary syphilis. Nearly all the vomer and nasal bones destroyed. Perforation about centre of hard palate on median line about three-fourths of an inch in diameter. The maxilla anterior to perforation had been partly destroyed by disease, the remainder had been removed; also the incisors on the right; the incisor, cuspid, and first bicuspid on the left. (Fig. 3.)

Three of the incisors on the lower jaw had never developed. A rubber plate was made bridging across the perforation fitting the lingual surface of the teeth, with teeth attached to supply the missing ones.

1874. Male, aged sixty. Syphilis acquired. Perforation two by one and one-fourth inches in diameter with a circumference of five and one-fourth inches, through hard and extending into soft palate. Nearly all the vomer and nasal bones destroyed. About ten or twelve years previous to 1874, the perforation about the size

Fig. 3.

of a pea, patient commenced using plugs of cotton renewed daily; the expansion of the cotton increased the dimension of the opening. (Fig. 4.)

Fig. 4.

These cotton plugs restored articulation and prevented liquids passing out the nose.

Plate made of hard rubber bridging across the perforation, fitting lingual surface of the teeth, clasped to first bicuspid on the left (second one missing) and around last molar on right side. (Fig. 5.)

Fig. 5.

To obtain an impression for the obturator in a congenital case *no* special tray is required, provided it is not unnecessarily large. The impression should be taken in plaster of Paris of the entire hard palate including the teeth. There is no necessity of its extending beyond the posterior border of the hard palate, *no impression* of the fissure of the soft palate being required.

When the hard palate is perforated *no* impression above the fissure is required; exception is made if teeth are lacking or incapable of giving support.

In taking an impression above the fissure in the hard palate (congenital cases), as a rule, fill the entire cavity with modelling composition, marking the lower surface with ridges or holes.

After this has hardened, and without removing it, procure an impression in plaster of Paris of the entire upper jaw, including the teeth.

Remove the plaster impression, then the composition, placing the latter in position on the plaster. An accurate impression is thus obtained.

Before this procedure make a careful examination of the teeth, and if none are missing nor any space between them, wedge apart the second bicuspid and first molar on each side for the purpose of clasping. These, when strong and free from decay, should always be used.

The clasps (Fig. 6) to be made of clasp gold, fitting the buccal

Fig. 6.

surface of both bicuspids and molars, extending between the teeth and vulcanized in the rubber.

The plate should cover the roof of the mouth, fitting accurately the lingual surface of the teeth posterior to the first bicuspids in all cases where the fissure does not extend through the hard palate.

Fig. 7.

If the cleft involves the hard palate, the plate should cover the opening, not extending above the floor of the nasal cavities, except in case of needed support. This, when required, may be accomplished by allowing the plate to extend above and beyond the fissure.

The length of the fissure may be obtained by a strip of gutta-percha about one-fourth of an inch wide, held in position with tweezers at the median line of the anterior end of the fissure, and moulded to conform to the curve of it.

Clasp gold (No. 23 gauge) is cut in length and bent in form to correspond to the gutta-percha. This should be vulcanized in or attached to the plate, extending along the median line of the fissure and elevated about one-eighth of an inch above it. (Fig. 7.)

Fig. 8 shows a gold plate with clasps, and the gold attachment soldered to it.

Fig. 8.

A preparation of three parts beeswax, two of paraffin, should be moulded around this gold attachment, inserted, then remoulded, then reinserted, until an obturator is formed about one-eighth of an inch in thickness, and, if the muscles and tissue will admit, one-eighth of an inch wider than the fissure above and posterior to it, extending backward nearly to the pharynx and downward, terminating at the extreme end of the fissure. This will bring the obturator nearly in contact with the dorsum of the tongue. If, in modelling, the tissues become irritated and nausea is produced, it may be delayed a few days, or the palate or pharynx may be sprayed with a solution of cocaine.

This waxed obturator should be worn by the patient a few hours; the temperature of the mouth will soften the wax sufficiently to admit of the muscles adapting themselves to it.

Reproduce this in hard rubber. In all cases retain one-third of the gold for the better attachment of the obturator to the plate, and bend in the form of a hook or drill holes through it. To avoid porosity and weight the obturator is sometimes made hollow. To do this impressions should be taken in plaster of Paris of the wax obturator and reproduced in type or Babbitt metal.

Figs. 9 and 10 show how type metal may be embedded in plaster of Paris in a square flask, and both plate and obturator reproduced. (Fig. 11.)

Fig. 9.

The process is as follows: First pack the space around the gold in the ordinary way. Two sheets of rubber are then cut to correspond to size of the mould. The edges are moistened with a preparation of rubber and naphtha or rubber and chloroform, by which they are joined firmly together; before the last opening is closed a small quantity of carbonate of ammonia is put inside, which, when subjected to heat in vulcanizing, will cause the rubber to expand and fill out the mould. This must be of metal to resist the pressure due to expansion.

The surface of the mould should be soaped before the rubber bag is placed in position for vulcanizing, to prevent rubber adhering.

When the mould is opened after vulcanizing, it will contain a perfect hollow bulb, with no marks of the places where the pieces

FIG. 10.

of rubber were joined, with the exception of a slight ridge made by the mould.

FIG. 11.

This bulb is finished in the same manner as a rubber plate.

Fig. 12 represents the appliance in position. A, A, the position of obturator above the fissure.

1877. The next case was a boy, aged six weeks. Congenital; fissure extending through hard and soft palate; hare-lip on right side. Patient etherized for purpose of operating on lip; previous

Fig. 12.

to this operation an impression was taken in plaster of Paris of hard and soft palate including the fissure. (Fig. 13.)

In cases where the fissure involves the hard and soft palate it is advisable, as soon as possible after the development of the deciduous teeth, to make an appliance bridging across the cleft of the hard palate.

This will prove of benefit both in mastication and articulation, and will prevent liquids from passing out of the nose, and assist in acquiring the habit of normal breathing. The appliance may be completed when the child is old enough to be controlled.

When there is a fissure involving only the soft palate, an obturator should be adapted at an early age (six or seven years). While this appliance will only be temporary (until the development of the permanent teeth), it will greatly aid the acquiring of proper articulation.

1877. Male, aged twenty-eight. Congenital. No hare-lip.

Fissure extending from a point corresponding to the second bicuspid, along the median line through the soft palate.

The appliance of hard rubber, with the bulb hollow. The plate covered the roof of the mouth posterior to the cuspid teeth extending back to the second molars, and fitting the lingual surface of the bicuspids and first molars. (Illustrated in Figs. 11 and 12.)

Previous to this, water and alcohol had been used for inflating the bulb with variable results. After experimenting, carbonate of ammonia was substituted, thus rendering the inflation certain.

FIG. 13.

In using plaster of Paris, it was found not to be strong enough to resist the great pressure (caused by the expansion of the ammonia) in vulcanizing, and type metal was substituted.

This patient had worn for a number of years, previous to 1877, a soft rubber palate. From eight to ten duplicates were required each year, as the secretions of the mouth and fermentation of food-particles made them unfit for use after a month or six weeks wear. The condition of the throat and general health of the patient was such that his physician advised him to abandon the use of soft rubber.

For similar reasons it has been found necessary to substitute hard for soft rubber in several cases.

After inserting the hard rubber obturator there was a decided improvement in articulation. His throat and general health was better, and the appliance has been worn continuously for twelve years without any annoyance or additional expense.

1877. Male, aged forty. Congenital. No hare-lip. Fissure of soft palate only; first bicuspid and first molar on the right side, lateral incisor, bicuspids, and first molar on the left had been extracted.

Plate and obturator made of hard rubber. The plate to which was added the first bicuspid on the right; the lateral incisor and bicuspids on the left were clasped on the right side to the second bicuspid, on the left to the second molar.

Fig. 14.

In this case the obturator was very thin, being only about one-eighth of an inch in thickness through the centre. The edges were rounded and somewhat thicker than the middle part. In making these thin obturators, round, smooth edges are necessary, as they are less liable to irritate.

This patient was a car-driver, a man of ordinary intelligence. The appliance was the first he had ever worn, and without any special instruction he learned rapidly to articulate distinctly.

After wearing the appliance a few months, the patient reported with great satisfaction that his improved articulation was a source of much comment among his acquaintances.

1877. B., aged thirty-seven. Congenital fissure, commencing centre of hard, extending along the median line through the soft palate. Hare-lip operated on a few weeks after birth. Only teeth remaining were the cuspid and first bicuspid, left side. For nine years previous had worn a gold plate with a soft rubber palate attached. This appliance was discarded as the decomposing of the rubber caused irritation and an offensive odor; the procuring of duplicates was a source of annoyance and expense.

Gold plate with teeth attached, to replace missing ones, was made with a hard rubber hollow bulb attached.

Fig. 15.

The appliance was worn with comfort and improved articulation.

1878. Male, aged nineteen. Congenital. Hare-lip on right side; operated on a few weeks after birth; fissure extending through hard and soft palate. Lateral incisor right side missing; supernumerary tooth back of cuspid tooth. Three attempts had been made to close the fissure by operations. (Fig. 14.)

Obturator made of hard rubber and hollow. The benefit derived from wearing the appliance was greater than had been anticipated, and the improvement in articulation marked.

1881. Female, aged eighteen. Hare-lip on right side operated on a few months after birth. Congenital. Had come from England to have an appliance made. The lip was imperfect, the nostril enlarged. Occlusion of the teeth very imperfect. First bicuspids had been extracted; the central and lateral incisors on right side never developed. The articulation was so imperfect that she could only be understood with difficulty. (Fig. 15.)

The appliance was of hard rubber with one lateral incisor and the bicuspids attached, bridging across the opening, extending back to the second molars fitting the lingual surface of the teeth, and secured with clasps.

The obturator was about one-eighth of an inch in thickness with the edges rounded. Shortly after the appliance was fitted the patient placed herself under the care of Miss Warren, of New York City, a teacher skilled in instructing deaf mutes to articulate. The results were wonderful. Her articulation became nearly perfect.

Seven years after the appliance was made she returned to this country for the purpose of having a duplicate. This was made the same as the first, with the exception that it was about one-fourth of an inch longer.

The patient was more than ordinarily grateful, for after wearing the appliance, her improved articulation enabled her to enter society, from which she had previously been debarred.

1879. Male, aged fifteen. Congenital fissure of soft palate extending into the palate bone.

A plate was made of hard rubber covering the roof of the mouth posterior to the first bicuspids, extending back to the twelve-year molars, fitting the lingual surface of the teeth and clasped to the six-year molars.

The obturator also of hard rubber, solid, about one-fourth of an inch in thickness, rounded at the edge, and attached to the plate at the junction of the hard and soft palate by a hinge. This hinge was used to bring into action the levator palati and the superior constrictor muscles, thus cutting off nasal communication at will, and was made of platinum and iridium, these metals being less likely to corrode.

This advantage was more than counterbalanced by the annoyance caused by food-particles becoming lodged in the hinge-joint, impeding its free movement. Its use has been entirely discontinued, except in cases where a full set of artificial teeth is worn, as the action of the hinge, in speaking, eating, or drinking, prevents displacement of the plate.

1881. Male, aged thirty-three; congenital fissure; no hare-lip. Fissure extending along the median line from a point corresponding to the twelve-year molars, through the soft palate. (See Fig. 7.)

This patient was wearing an appliance, consisting of a metal bulb with a plate as follows: "The roof of the mouth being very high in the centre, the cast was filled up at that point so as to bring the golden roof at a lower level and make the dome more symmetrical and better formed for enunciation. Subsequently the span above the gold was filled with vulcanite."

This form of plate had proved of no advantage in enunciation; on the other hand, the weight was increased; this increased weight and the imperfect clasping of the plate rendered it less firm and caused unnecessary wear on the teeth.

A new plate was made of twenty-carat gold (No. 27 gauge), fitting accurately the roof of the mouth from the second bicuspid, including the second molars on each side, fitting the lingual surface of the teeth, and clasped with a double clasp to the bicuspids and molars on each side. To this plate was attached the bulb previously worn.

About six months ago a new plate and obturator were made. The plate a fac-simile of the last one described. The obturator was of hard rubber, very thin, with rounded edges.

This appliance was made for a gentleman of high social standing, superior intelligence, and keen judgment.

The following communication, relating his experience, ought to carry greater weight and more positive proof of the comparative merits of different appliances than anything that can be said or written by the operator.

November 14, 1889.

Dr. Kasson C. Gibson:

My dear Sir,—In the year 1866 it was suggested to me that I see a dental specialist in reference to an appliance for defective palate. I did so, and the specialist made for me a *soft rubber appliance*, which I used continuously until 1875. In this year he made for me a *metal bulb*, which I have used continuously from October, 1875, until this autumn, 1889. In October, 1881, eight years ago, I placed myself in your care, finding great relief from the better attachment of the appliance to the teeth, a double clasp being used, giving firmness and not such wear on the teeth; in fact, previous to October, 1881, the appliance *never at any time* set snug and close to the mouth and teeth, so that the teeth were rapidly wearing and cutting away. During the past six months you have made and fitted for me an appliance of hard rubber; this I am using now. During all these years (being twenty-three) I have used each variety and have had full experience, sparing no time, money, or pains, to get the best results. For the first ten years I practised under an elocutionist, finding great benefit, and, at times, I still do the same.

As to the soft rubber palate, I was extremely glad to be relieved from it, because it is soft, flabby, lacking stiffness, making it difficult to use or pronounce some words clearly or distinctly, and after a few days of use becoming very disagreeable and offensive unless great attention was paid to it by boiling; in fact, two months was the limit of time comfortably to use one, and then a source of trouble in procuring new ones, which led me to vulcanize them myself in moulds made for me; this all kept up the cost, which I found was quite an item in a year.

The use of the bulb was much better, gave better control to the voice, clean and pleasant. But in my experience I found that there was *too much bulb*, it closed up too much, so that it muffled the voice, prevented the free passage of mucus, allowed food to sometimes get lodged on it, and was heavy.

In the last appliance I think the end desired has been practically reached. None of the difficulties experienced in the others are found in this. It is light in weight, clean, allowing food, mucus, and air to pass without hinderance, gives a good clear tone to the voice and easier pronunciation, so that *no one* could detect or imagine an appliance was in use, and it is a regret that it could not have been used in the beginning, twenty-three years ago, for I can see no reason why a person should not use hard rubber from the start.

Yours faithfully,

A Patient.

## Figures and Tables

**Fig. 1. f1:**
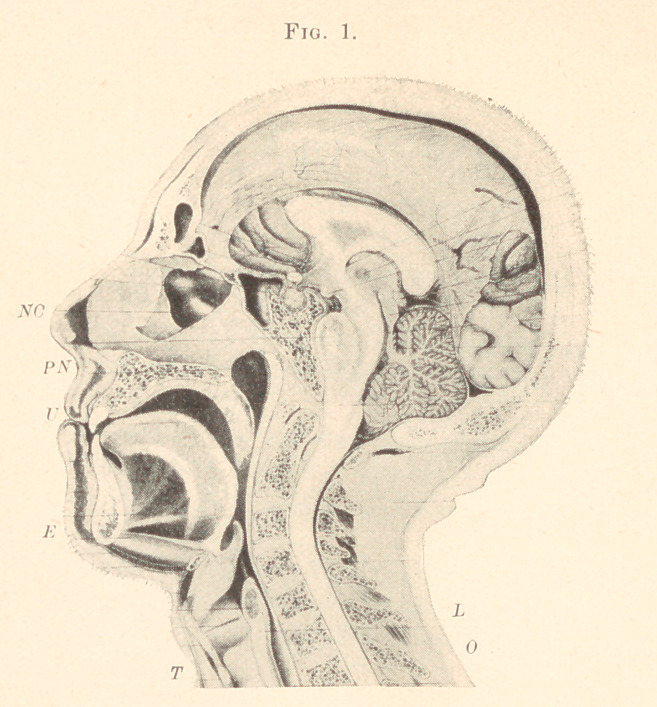


**Fig. 2. f2:**
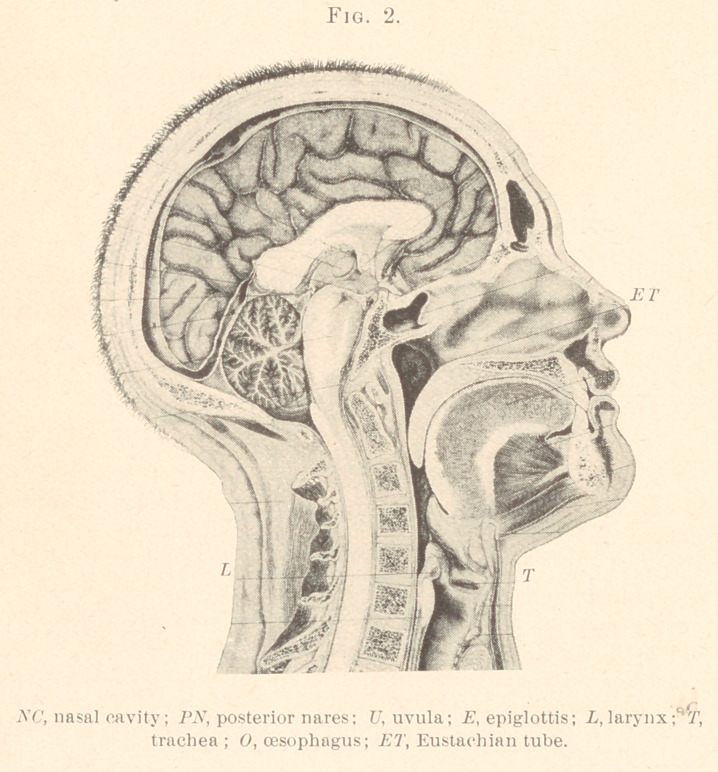


**Fig. 3. f3:**
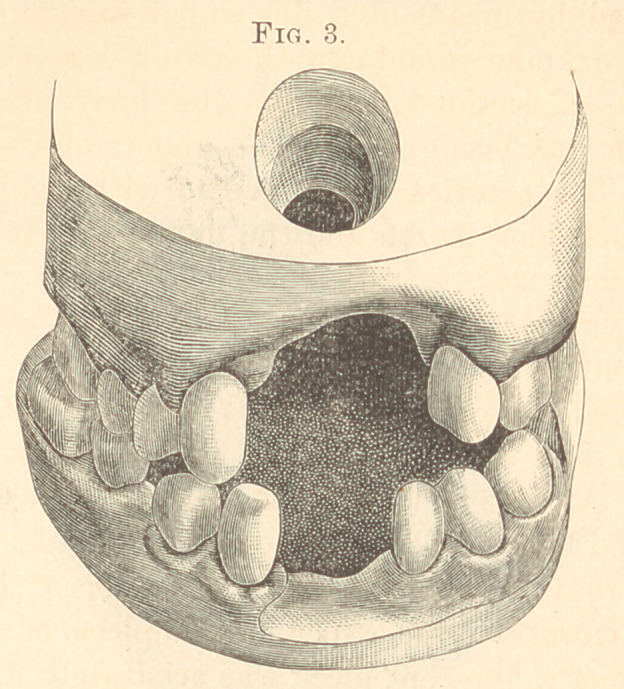


**Fig. 4. f4:**
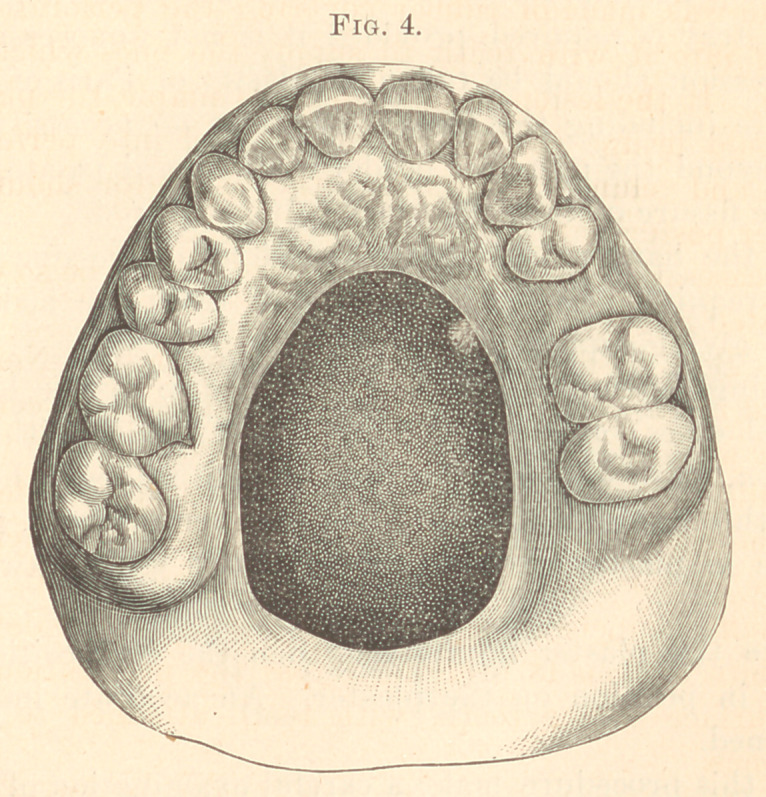


**Fig. 5. f5:**
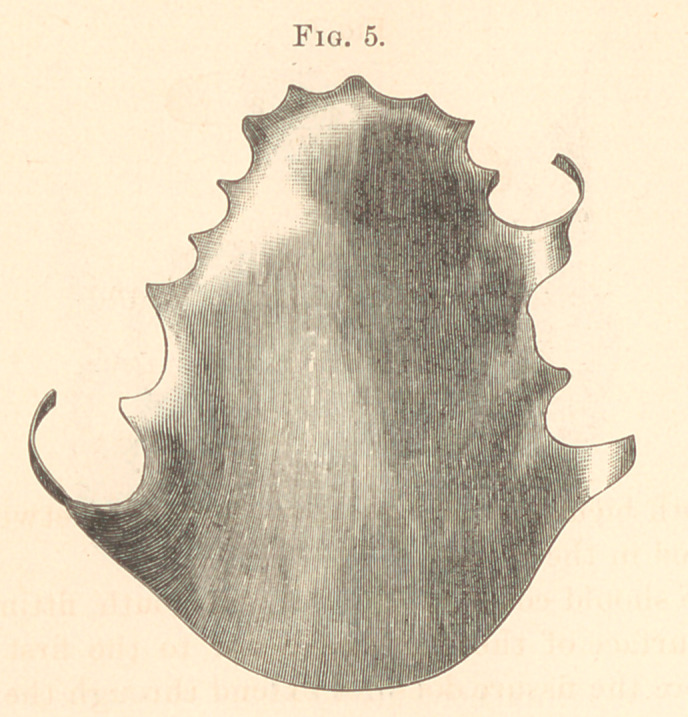


**Fig. 6. f6:**
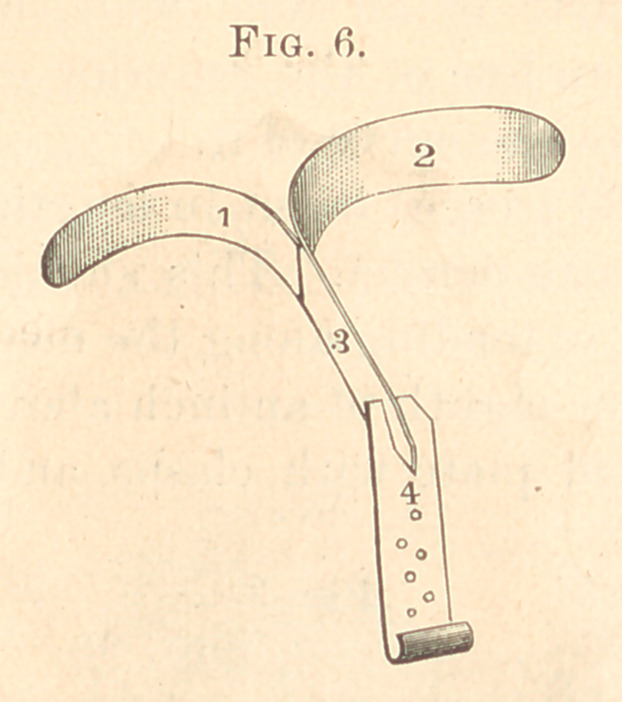


**Fig. 7. f7:**
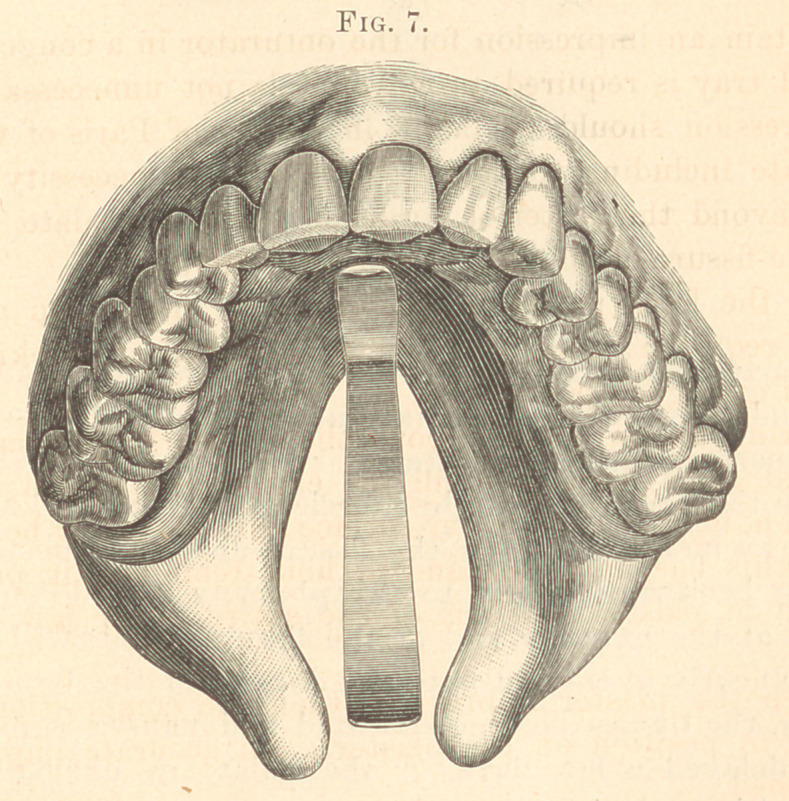


**Fig. 8. f8:**
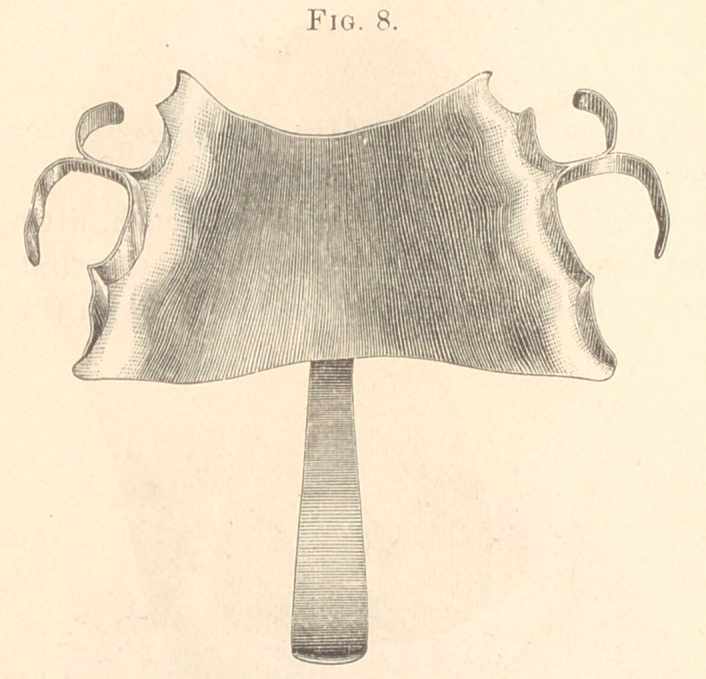


**Fig. 9. f9:**
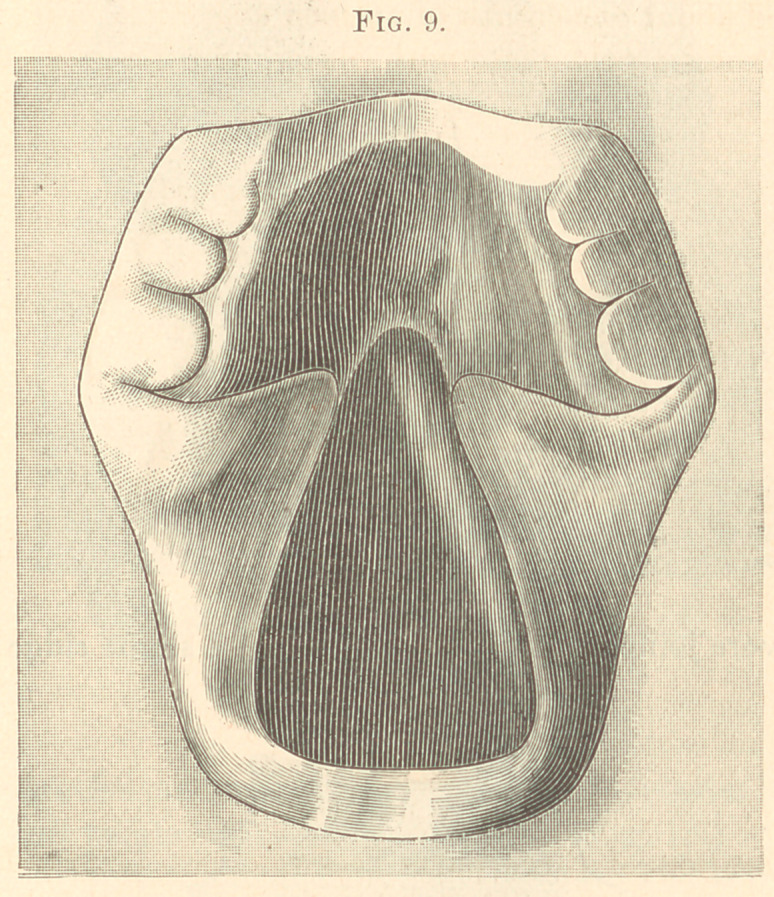


**Fig. 10. f10:**
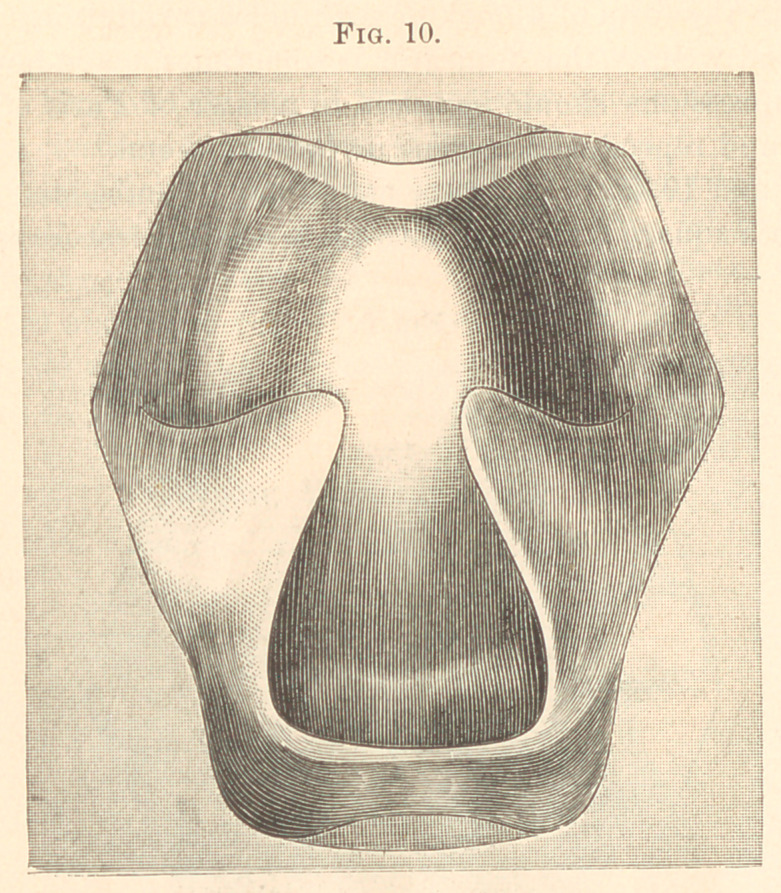


**Fig. 11. f11:**
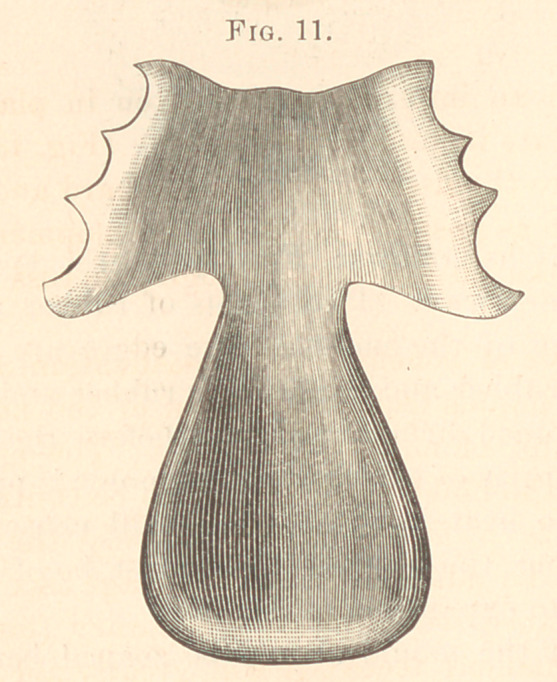


**Fig. 12. f12:**
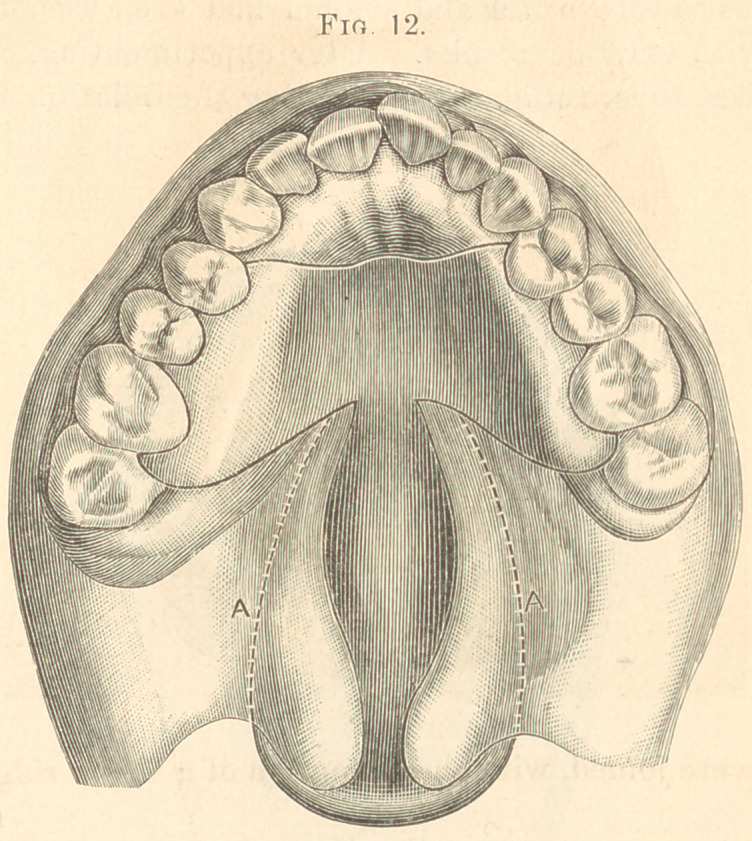


**Fig. 13. f13:**
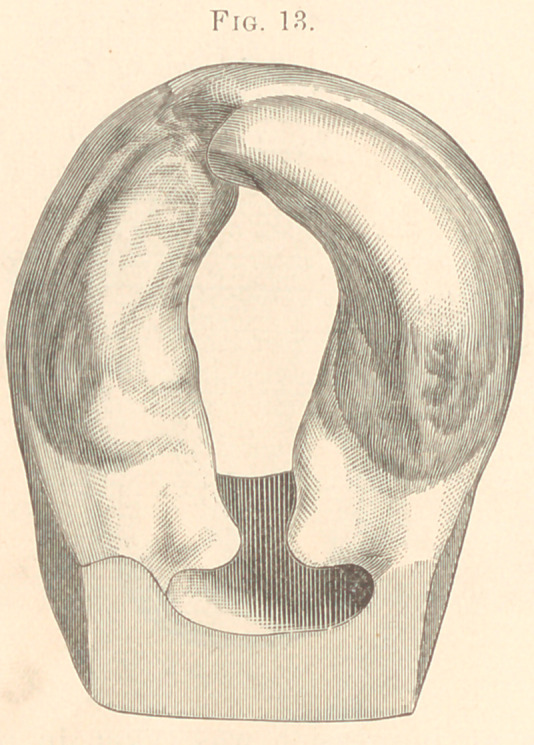


**Fig. 14. f14:**
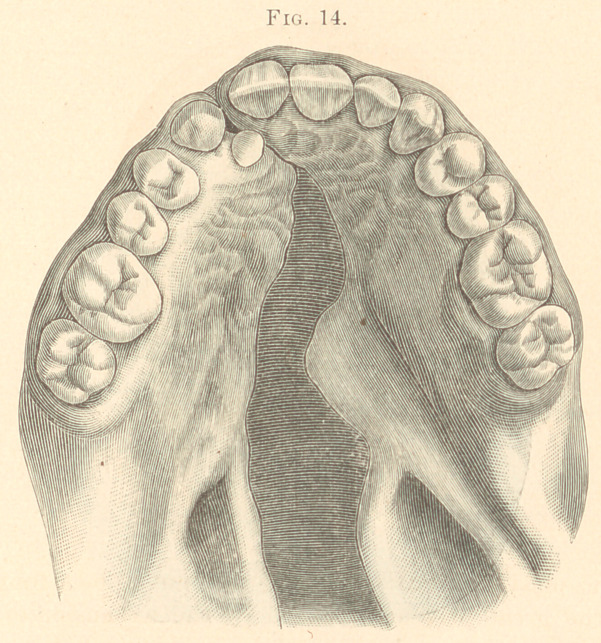


**Fig. 15. f15:**